# The Radcliffe Infirmary

**Published:** 1895-01-19

**Authors:** 


### THE RADCLIFFE INFIRMARY.

Our criticism and our advice regarding the management and status of the Radcliffe Infirmary have drawn forth the somewhat weak reply that the infirmary is not a University institution; that the University neither aids it with funds nor helps in its management; and that, in fact, the infirmary is but a city and county affair, inadequately supported by the villadom of North Oxford and by a county suffering profoundly from agricultural depression, and unable to provide funds. But surely that is the very centre of our complaint. Institutions, like individuals, must, when greatness is thrust upon them, either accept it with all its responsibilities or sink into insignificance, and it is idle for people living in a city like Oxford, with its University, its colleges, its professors of medicine and physiology, its splendid museum, and its school of anatomy, to say that their hospital is to be a mere county iufirmary, and not equal to Addenbrooke's Hospital, Cambridge.

To submit to be provincial is but to accept the inevitable in the case of ordinary county towns, but Oxford is not an ordinary county town; Oxford centres in the University, without which it would be dead, unprofitable, and unknown, and it should be recognised at once that to accept for the hospital the standard perforce accepted by some county infimaries is definitely to cut itself adrift from all the life and interests of the city of which it should be a part.

We are not concerned with the question of the actual medical teaching of medical students within the hospital walls. To say that students will always do their ward work in London, and that Oxford can never pretend to give the complete medical curriculum, may be true enough, but is no excuse for neglecting to put the hospital on any less perfect footing. What we say is that for Oxford, a centre of learning, a resort of students, a very hot-bed of professors, a place which aims especially at perfection and completeness of knowledge; to draw the line at mere knowledge and refuse to interest itself in the perfection of its practical application, and especially its application to the good of others, is an absurd position. Modern teaching of medicine in the University should be linked with modern hospital management in the infirmary.

How to ensure this is now the problem, and we hope that at the Quarterly Court of Governors, to be held on the 23rd of this month, some steps will be taken to lift the whole subject on to a higher platform, and to ask the mayor to call a public meeting to consider the matter in all its bearings.

There are four public interests which are concerned, the University, the city, the county, and the infirmary itself, in which those of the others should unite.

We entirely decline to admit that the University has no interest in the matter. To teach medicine in theory, and to allow the hospital in which it is reduced to practice to be anything short of the best is to cover itself with ridicule, and in any steps which are taken to introduce reforms into the management of the infirmary the University should play a part as well as the city and the county.

Let Oxford then bring the structure of its Radcliffe Infirmary into conformity with modern standards; let the inner management be modernised, always bearing in mind that it is a medical institution and that in its governance due power should be given to its medical officers; let a strong resident be appointed, who will speak with authority as to matters of internal discipline; let outside matters be entrusted to a skilled secretary, well acquainted with hospital finance; let the training of nurses be entirely dissevered from all housekeeping cares; and then there will be a possibility of the Radcliffe Infirmary taking the position it ought to take as a worthy helpmate to the University.

